# Amlodipine Reduces AngII-Induced Aortic Aneurysms and Atherosclerosis in Hypercholesterolemic Mice

**DOI:** 10.1371/journal.pone.0081743

**Published:** 2013-11-14

**Authors:** Xiaofeng Chen, Debra L. Rateri, Deborah A. Howatt, Anju Balakrishnan, Jessica J. Moorleghen, Andrew J. Morris, Richard Charnigo, Lisa A. Cassis, Alan Daugherty

**Affiliations:** 1 Saha Cardiovascular Research Center, University of Kentucky, Lexington, Kentucky, United States of America; 2 Department of Biostatistics, University of Kentucky, Lexington, Kentucky, United States of America; 3 Department of Molecular and Biochemical Pharmacology, University of Kentucky, Lexington, Kentucky, United States of America; Brigham and Women's Hospital, Harvard Medical School, United States of America

## Abstract

**Background:**

The purpose of this study was to determine effects of amlodipine, a dihydropyridine calcium channel blocker, on development of angiotensin II (AngII)-induced vascular pathologies.

**Methods and Results:**

Male LDL receptor -/- mice were infused with vehicle, amlodipine (5 mg/kg/d), AngII (1,000 ng/kg/min), or AngII + amlodipine for 4 weeks through osmotic pumps (n=10/group). Mice were fed a saturated fat-enriched diet for 1 week prior to pump implantation and during 4 weeks of infusion. Infusion of amlodipine resulted in plasma concentrations of 32 ± 2 ng/ml and 27 ± 2 ng/ml for mice in saline + amlodipine and AngII + amlodipine groups, respectively. This infusion rate of amlodipine did not affect AngII-induced increases in systolic blood pressure. Three of 10 (30%) mice infused with AngII died of aortic rupture, while aortic rupture did not occur in mice co-infused with AngII + amlodipine. Suprarenal aortic width and intimal area of ascending aortas were measured to define aortic aneurysms. In the absence of AngII infusion, amlodipine did not change suprarenal aortic width and ascending aortic area. Infusion of AngII led to profound increases of suprarenal aortic width (saline + vehicle versus AngII + vehicle: 0.86 ± 0.02 versus 1.72 ± 0.26 mm; P=0.0006), whereas co-infusion of AngII and amlodipine diminished abdominal dilation (1.02 ± 0.14 mm; P=0.003). As expected, AngII infusion increased mean intimal area of ascending aortas (saline + vehicle versus AngII + vehicle: 8.5 ± 0.3 versus 12.5 ± 1.1 mm^2^; P=0.001), while co-infusion of AngII and amlodipine ablated dilation of the ascending aorta (8.6 ± 0.2 mm^2^; P=0.03). Co-administration of amlodipine also significantly attenuated AngII-induced atherosclerosis in the thoracic region as quantified by percent lesion area (AngII + vehicle versus AngII + amlodipine: 5.8 ± 2.1 % versus 0.3 ± 0.1%; P=0.05).

**Conclusions:**

Amlodipine inhibited AngII-induced aortic aneurysms in both the abdominal and ascending regions, and atherosclerosis in hypercholesterolemic mice.

## Introduction

 Aortic aneurysmal and atherosclerotic diseases may occur coincidently in humans [1-5]. Excessive stimulation of the renin angiotensin system (RAS) has been implicated in many human cardiovascular diseases, including aortic aneurysms and atherosclerosis [6,7]. There are substantial experimental data that excessive RAS activation also promotes these diseases in animal models. AngII infusion into LDL receptor -/-, apoE -/- or normolipidemic mice induces development of aneurysms in the suprarenal region of the abdominal aorta [8-11]. More recently, it has been noted that AngII infusion also promotes pronounced dilation of the thoracic aorta, which is restricted to the ascending region [12,13]. In addition to promoting formation of aortic aneurysms, many studies have reported that chronic subcutaneous infusion of AngII into mice augments development of atherosclerosis in both apoE -/- and LDL receptor -/- mice [8,9,14]. 

 Since AngII infusion into hypercholesterolemic mice promotes diverse aortic pathologies, this model permits simultaneous determination of effects of an intervention on abdominal and thoracic aortic aneurysms as well as atherosclerosis. Several studies have demonstrated that interventions can have differential effects on abdominal aortic aneurysms and atherosclerosis. These include pharmacological interventions with doxycycline, bosentan, and pioglitazone [15-17], and surgical interventions such as orchidectomy [18]. Genetic manipulations have also provided divergent effects including whole body genetic deletion of interferon-gamma, CXCL10, receptor-associated protein, or Rag-1 [19-21], and cell-specific deletion of PPAR gamma or AT1a receptors [17,22]. In contrast, several interventions have similar effects on these pathologies including pharmacological inhibition or whole body genetic deficiency of AT1a receptor [23,24], an AT2 receptor antagonist [23] and whole body deficiency of CCR2 [12]. Whole body deficiency of AT1a receptor or CCR2 also decreased AngII-induced thoracic aortic aneurysms [12,22]. Findings from these studies implicate that the aortic pathologies in aneurysms and atherosclerosis may be attributed to a combination of common and distinctive mechanisms.

 Anti-hypertensive drugs, including calcium channel blockers, are commonly prescribed for hypertensive patients who frequently also have aortic aneurysmal or atherosclerotic diseases. Although one study indicates that this class of drugs may have detrimental effects on abdominal aortic aneurysms in humans [25], calcium channel blockade has been shown to decrease atherosclerosis in humans [25-30]. However, this has not been determined in prospective studies. The purpose of this study was to determine effects of a commonly used calcium channel blocker, amlodipine, on aortic pathologies in a mouse model that develops aortic aneurysms and atherosclerosis simultaneously.

## Materials and Methods

### Ethics Statement

 Recommendations from the Guide for the Care and Use of Laboratory Animals (National Institutes of Health) were followed. All procedures were approved by the University of Kentucky Institutional Animal Care and Use Committee (Protocol # 2006-0009). Mice were examined daily for signs of distress, dehydration, lack of weight gain, or bilateral hind leg paralysis. No adverse effects were noted during this study. Mice were terminated by overdose of a ketamine/xylazine mixture.

### Mice

 Male low-density lipoprotein (LDL) receptor -/- mice (stock # 002207; > N10 background into the C57BL/6 background) were purchased from the Jackson Laboratory (Bar Harbor, ME, USA). Mice were housed in individually ventilated cages with negative air pressure (Allentown Inc; Allentown, NJ, USA). Filtered drinking water by reverse osmosis system was provided ad libitum. Normal mouse diet (Global 18% protein rodent diet; Diet # 2918; Harlan Teklad; Madison, WI, USA) was fed. Light and dark cycle of the room was 14 hours of light and 10 hours of dark. Ambient temperature ranged from 20 to 23°C and humidity was 50-60%. Necropsy was performed on mice that died during AngII infusions within 12 hours of expiration. Systolic blood pressure was measured by a tail cuff based technique using Coda 8 machines (Kent Scientific, Torrington, CT, USA) as described previously [31].

### Diet and Drug Administration

 To induce hypercholesterolemia, mice were fed a diet enriched in saturated fat (milk fat 21% wt/wt) and 0.2% wt/wt cholesterol (Diet # TD.88137; Harlan Teklad) 1 week prior to osmotic mini-pump implantation and throughout the 4 weeks of infusions.

 Four groups (n=10 per group) of mice were infused with: 1. saline + vehicle [50% dimethyl sulfoxide (DMSO)]; 2. saline + amlodipine (5 mg/kg/day); 3. AngII (1,000 ng/kg/min) + vehicle (50% DMSO); and 4. AngII + amlodipine. For osmotic pump preparation (Durect Corporation; Cupertino, CA, USA), AngII (1,000 ng/kg/min; Cat # H-1705; Bachem; Torrance, CA, USA) was dissolved in saline. AngII and saline were infused using Alzet model 1004. Amlodipine besylate (5 mg/kg/day; Cat # A5605; Sigma-Aldrich; St. Louis, MO, USA) was dissolved in 50% DMSO. Amlodipine and vehicle (50% DMSO) were infused using Alzet model 2004. After anesthesia with ketamine/xylazine (90 and 10 mg/kg body weight, respectively), a Model 1004 pump and a Model 2004 pump were implanted subcutaneously into the flanks of each mouse [9]. A topical analgesic, LMX4 (Ferndale Laboratories; Ferndale, MI, USA), was used to provide relief from pain associated with surgery.

### Plasma Measurements

 Plasma cholesterol concentrations were measured using a commercially available enzymatic kit (Cat # Cholesterol E 439-17501; Wako Chemicals; Richmond, VA, USA) as described previously [32]. Plasma lipoprotein fractions were resolved by size exclusion chromatography and cholesterol concentrations were measured using the cholesterol enzymatic kit as described previously [32].

 Plasma amlodipine concentrations were measured by HPLC electrospray ionization tandem mass spectrometry using adaptation of a previously reported method [33]. Briefly, individual plasma samples (50 μl) were extracted with ethyl ether: dichloromethane (70:30). After centrifugation, the supernatant was evaporated to dryness. Dried samples were reconstituted in a mixture of ammonium formate/ methanol/acetonitrile (30:50:20; 50 µl). Samples (10 μl) were analyzed using a Shimadzu automated HPLC system with an Agilent Eclipse XDB C18 column (Agilent; Santa Clara, CA, USA) coupled with an AB Sciex 4000-Qtrap hybrid linear ion trap triple quadrupole mass spectrometer (AB Sciex; Framingham, MA, USA) that was operated in multiple reaction monitoring mode. Tizanidine (Cat # T6950; Sigma Aldrich) was used as an internal standard. Quantitation was accomplished by reference to an offline calibration generated using amlodipine that was independently quantitated by accurate mass measurements.

 Plasma renin concentrations were measured as described previously [34]. Briefly, individual plasma samples (8 μl) harvested with EDTA (1.8 mg/ml) were incubated with an excess of rat angiotensinogen at 37 °C for 30 minutes. The generated AngI was quantified by radioimmunoassay using a commercially available kit (Cat # 1553; DiaSorin; Stillwater, MN, USA).

### Quantification of Aortic Aneurysms and Atherosclerosis

 Aortas were dissected from the root to iliac bifurcation and placed in 10% neutrally buffered formalin overnight. After removal of adventitia, aortas were pinned and photographed. Maximal ex vivo diameters of suprarenal aortas were measured with ImagePro Plus software (Media Cybernetics; Rockville, MD, USA) to quantitate AAAs [11,35]. Subsequently, aortas were cut open longitudinally, secured with pins, and photographed. To determine formation of thoracic aortic aneurysms, dilation of ascending aortas was quantified by measurement of intimal area of the ascending aortic region [12]. Atherosclerosis was quantified on the intimal surface of the aorta by an en face technique as described previously [36,37].

### Histological and Immunohistochemical Staining

 Aortic tissues were fixed with 4% paraformaldehyde, and then sectioned on a cryostat at a thickness of 10 µm. Sections were histologically stained using Verhoeff’s hematoxylin to visualize the integrity of elastin. Immunohistochemical staining was performed as described previously [38]. Smooth muscle cells were detected using smooth muscle α-actin antibody (2 μg/ml; Abcam, cat# ab5694) and macrophages using an CD68 antibody (5 μg/ml; Serotec, cat# MCA1957GA). Positively reactive areas were visualized via application of an ABC kit (Vector) and subsequent detection with AEC chromogen (Vector). Images were captured using a Nikon Eclipse E600 scope and a Nikon DXM1200F digital camera. 

### Statistical Analyses

 Data for continuous variables are summarized as means ± standard error of means (SEM). Version 9.2 of SAS (SAS Institute, Cary NC) was used for data analysis (SAS Institute Inc.; Cary, NC, USA). Effects of AngII and amlodipine were assessed by two-way analysis of variance (ANOVA) if normality and equal variance assumptions were satisfied (or if violations of these assumptions could be addressed by transforming the data or reweighting the observations) and were assessed posthoc by nonparametric Kruskal-Wallis test otherwise. P≤0.05 was considered significantly different.

## Results

### Characteristics of Study Mice

**Figure 1:**
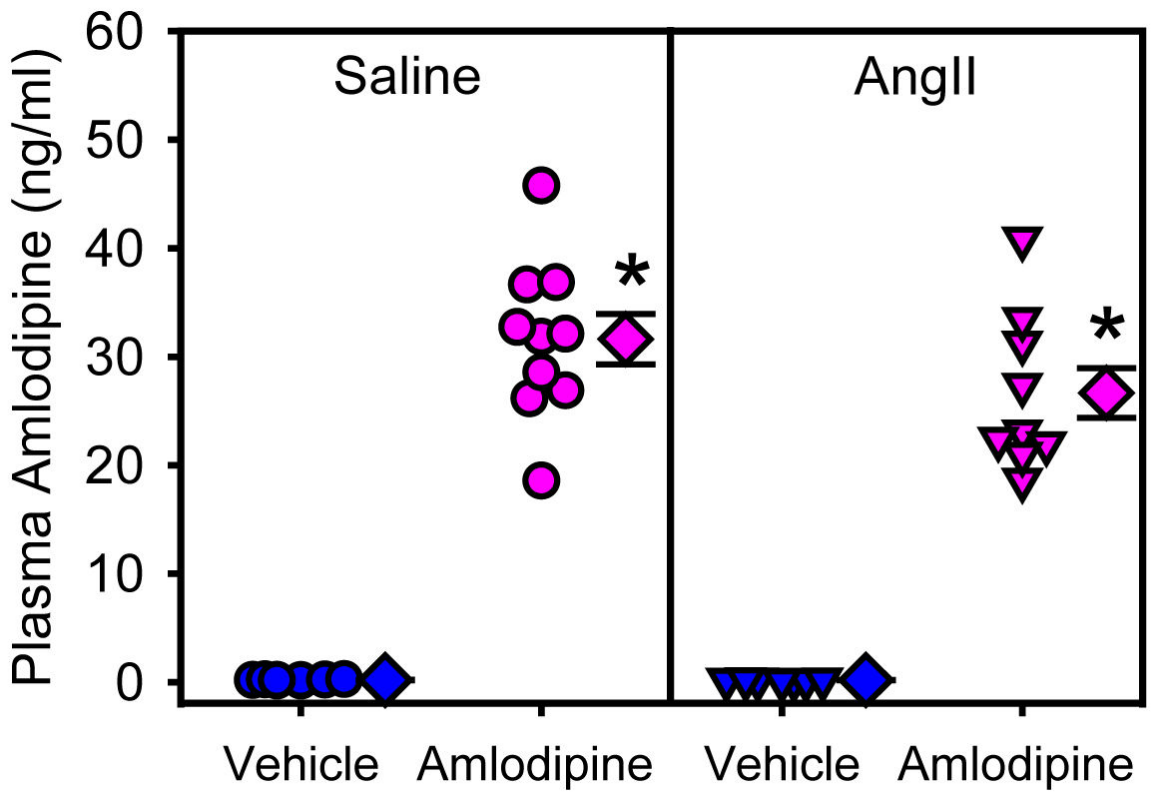
Plasma concentrations of amlodipine. Plasma concentrations of amlodipine were measured at the end of study. Circles and triangles represent individual mice, diamonds are means of groups, and error bars are SEMs. * denotes P<0.0001 comparing amlodipine groups with saline + vehicle and AngII + vehicle groups by two way ANOVA.

 Amlodipine was infused subcutaneously through osmotic pumps to maintain a constant plasma concentration throughout the study. Plasma concentrations of amlodipine were not significantly different between the groups that were co-infused with either saline or AngII (32 ± 2 ng/ml and 27 ± 2 ng/ml, respectively), while the drug was not detectable in saline + vehicle or AngII + vehicle infused mice (Figure 1). Amlodipine administration had no effect on body weight, plasma cholesterol concentrations, and plasma lipoprotein-cholesterol distributions in mice infused with either saline or AngII (Table 1). Amlodipine also had no significant effect on systolic blood pressure in mice infused with AngII (150 ± 3 versus 148 ± 4 mmHg for AngII + vehicle versus AngII + amlodipine-infused mice, respectively). AngII infusion significantly decreased plasma renin concentration (P=0.005); however, addition of amlodipine significantly increased plasma renin concentration (P<0.0001; Table 1). 

**Table 1:** Characteristics of study mice.

Infusions	Saline		AngII	
	Vehicle	Amlodipine	Vehicle	Amlodipine
n	10	10	7*	10
Body weight (g)	28.0 ± 0.5	27.6 ± 0.5	26.9 ± 0.6	26.9 ± 0.5
Plasma cholesterol (mg/dl)	1038 ± 41	949 ± 41	1168 ± 49	1119 ± 41
Plasma renin (ng/ml/30 min)	3.0 ± 0.3	8.5 ± 1.3^†^	1.1 ± 0.1^#^	5.2 ± 1.1^†#^

Body weight, plasma cholesterol and renin concentrations were measured after 28 days of infusions. Values are represented as mean ± SEM. * Ten mice were initiated in the study, but 3 succumbed to aortic rupture, so data were derived from 7 surviving mice. ^†^ P<0.0001, compared to vehicle within saline or AngII groups. ^#^ P=0.005, compared to saline infusion within vehicle or amlodipine groups.

### Amlodipine Prevented Development of AngII-induced Aneurysms in Both Abdominal and Ascending Aortic Regions

**Figure 2:**
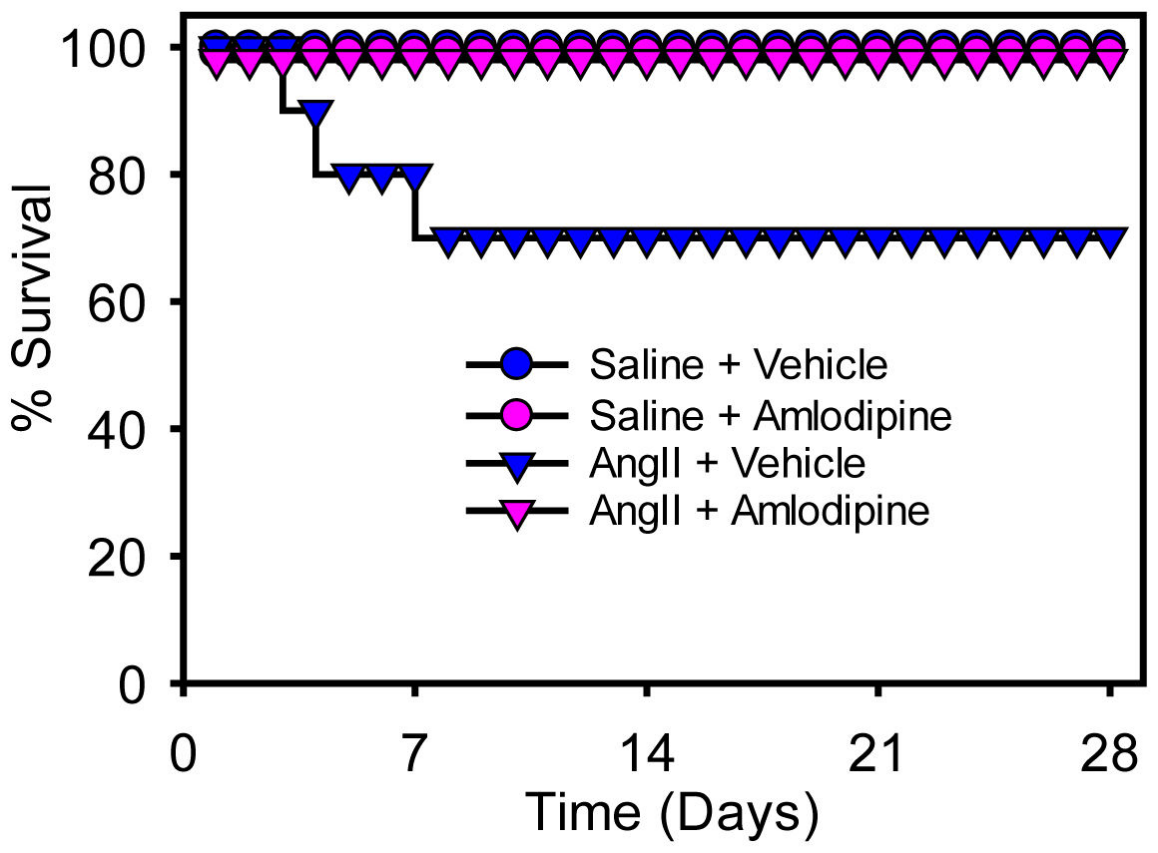
Survival curve for study mice. The survival curve for the four groups of mice during the infusion interval. Mice infused with AngII + vehicle had 3 deaths (occurred on days 3, 4, and 8) due to aortic rupture, while no deaths occurred in the other groups.

 Three of 10 mice infused with AngII died of aortic rupture within 10 days after initiating AngII infusion, while no aortic ruptures occurred in the other 3 groups as shown in Figure 2. 

**Figure 3:**
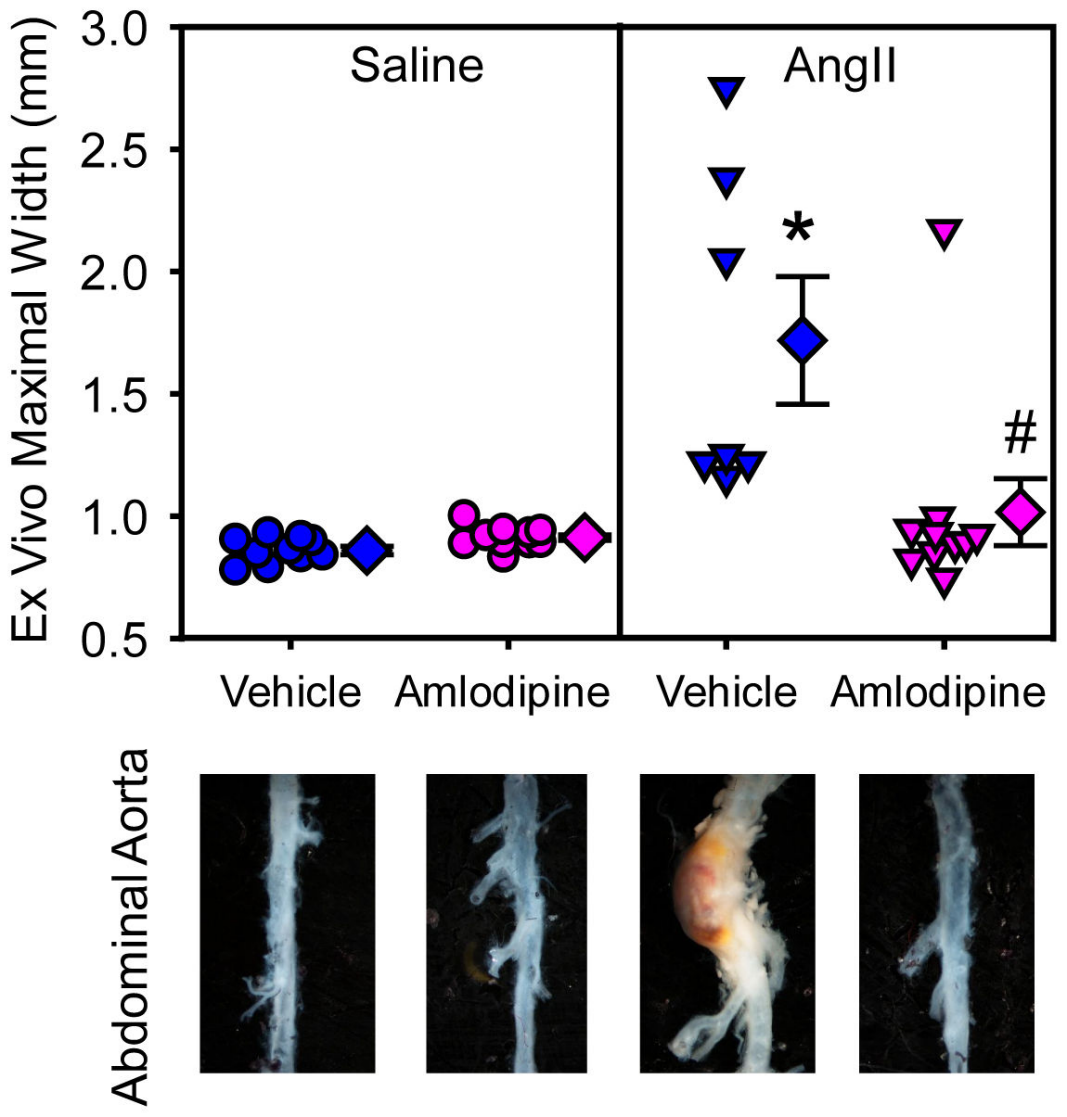
Amlodipine ablated AngII-induced abdominal aortic aneurysms. *Ex* *vivo* suprarenal aortic diameter was measured after termination. Circles and triangles are individual measurements, diamonds means of the groups, and error bars SEMs. Representative images of aortas are shown below the graph. Statistical analyses were performed using nonparametric Kruskal-Wallis test. * denotes P=0.0006 for comparison between AngII + vehicle and saline + vehicle, and ^#^ denotes P=0.003 for comparison between AngII + amlodipine and AngII + vehicle groups.

**Figure 4:**
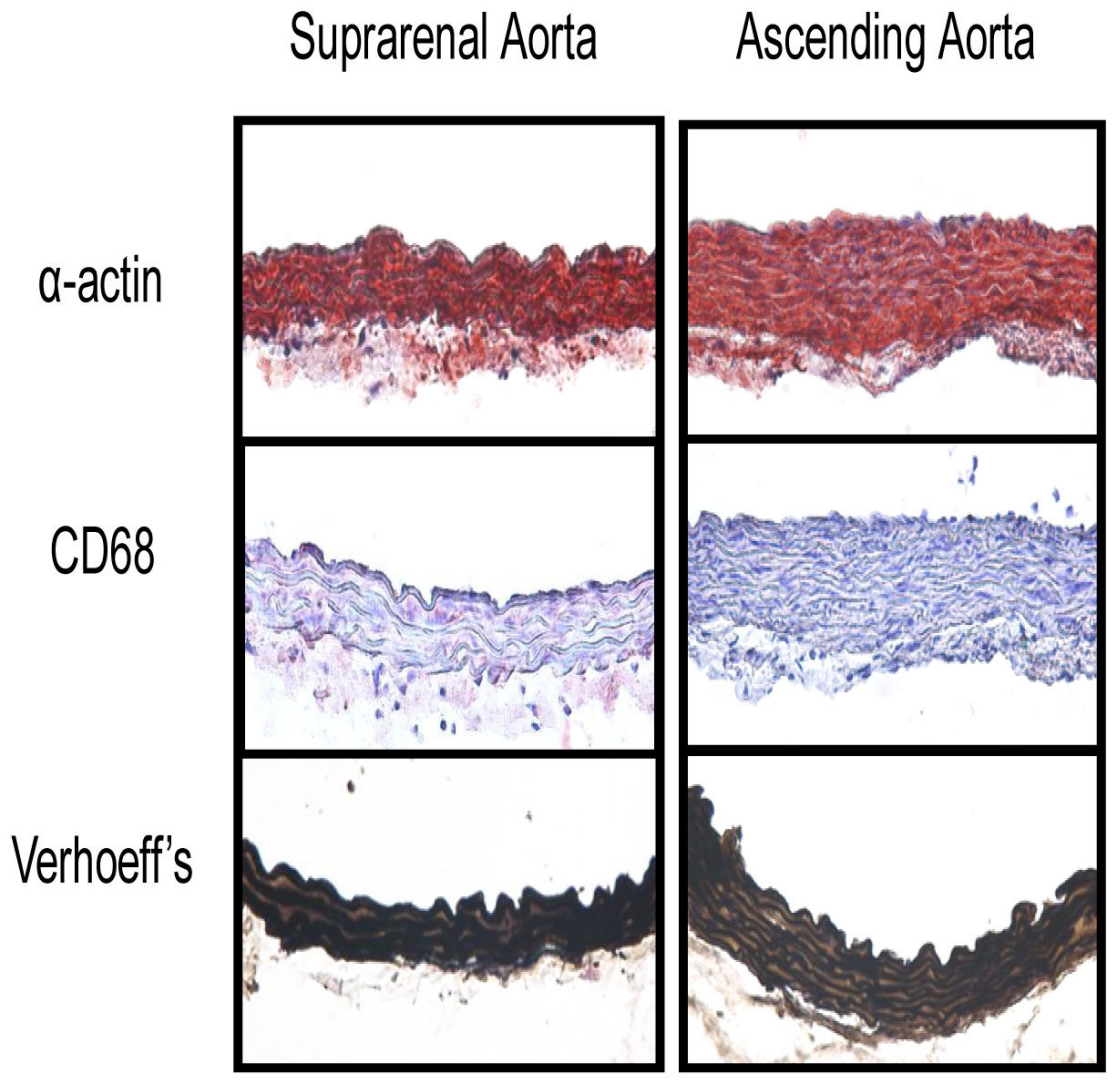
Amlodipine preserved normal aortic structure in the suprarenal and ascending regions. Tissue sections from AngII-infused mice also infused with amlodipine were immunostained with alpha actin and CD68 to detect smooth muscle cells and macrophages, respectively. Positive reactivity is show by intense red color. Verhoeff’s hematoxylin staining was performed to facilitate visualization of elastin.

 To determine effects of amlodipine on development of AAAs, maximal diameters of suprarenal aortas were measured ex vivo. Amlodipine had no effect on suprarenal aortic width in saline-infused mice. As described previously, infusion of AngII led to profound increases of maximal aortic width (saline + vehicle versus AngII + vehicle: 0.86 ± 0.02 versus 1.72 ± 0.26 mm; P=0.0006). Amlodipine administration significantly attenuated AngII-induced abdominal aortic dilation (1.02 ± 0.14 mm; P=0.003; Figure 3). Aortas from AngII-infused mice administered with amlodipine were grossly normal as demonstrated by histological analysis (Figure 4). To determine whether any pathological changes were discernible, aortic tissues were sectioned and immunohistochemically and histologically stained. As the example shown in Figure 4, suprarenal aortas from amlodipine infused mice were grossly normal with consistent smooth muscle α-actin immunostaining, intact elastin fibers in the media, and no accumulation of macrophages. 

**Figure 5:**
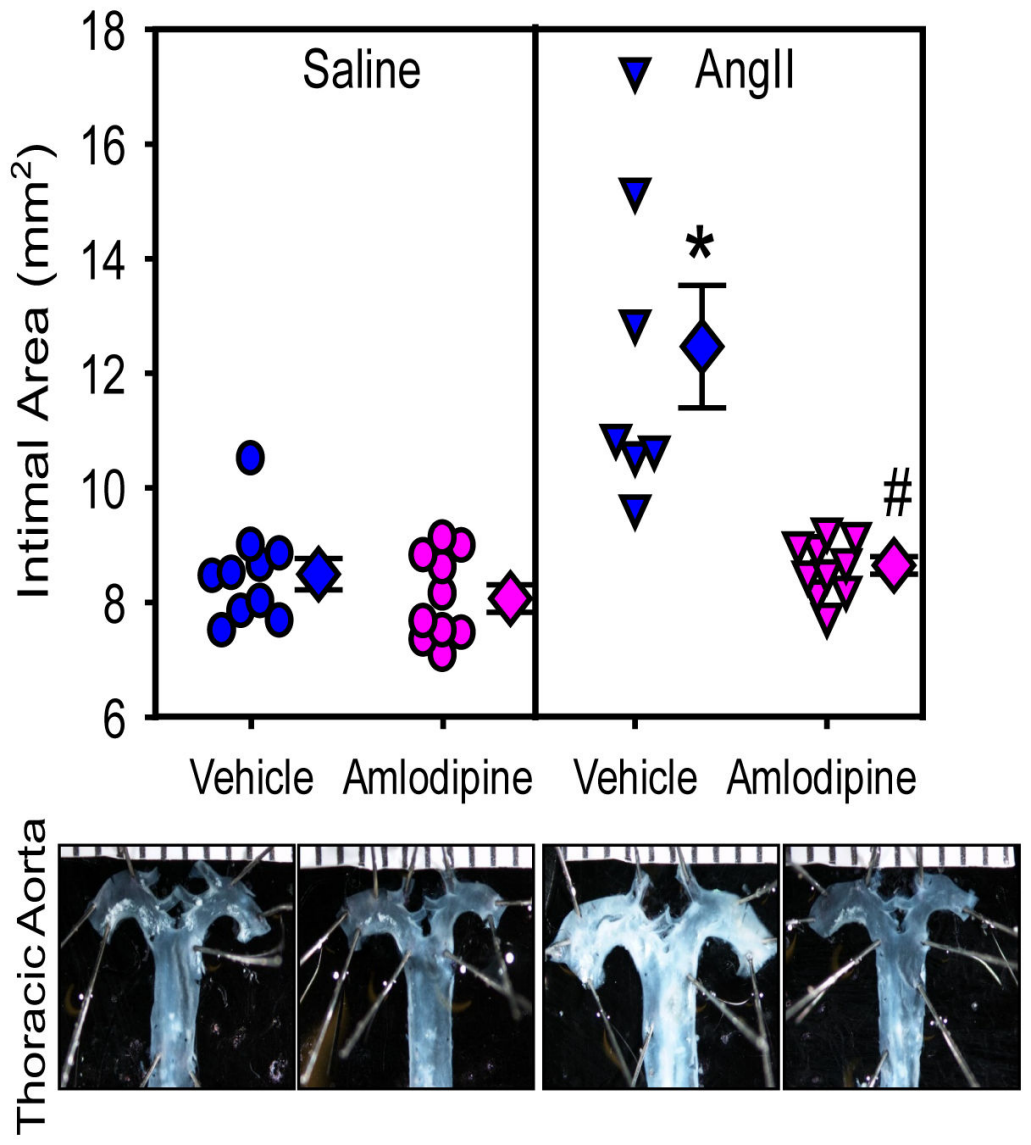
Amlodipine attenuated AngII-induced ascending aortic aneurysms. Ascending aortic areas were measured using an en face method. Circles and triangles are individual measurements, diamonds are means of groups, and error bars are SEMs. Representative images of aortas are shown below the graph. Statistical analyses were performed using nonparametric Kruskal-Wallis test. * denotes P=0.001 for comparison between AngII + vehicle and saline + vehicle groups, and ^#^ denotes P=0.03 for comparison between AngII + amlodipine and AngII + vehicle groups.

 En face measurement of intimal surface area of the ascending aorta was used as an index to determine ascending aortic dilation [12]. Administration of amlodipine had no effect on ascending aortic area in saline-infused mice. AngII infusion significantly increased mean intimal area of ascending aortas (saline + vehicle versus AngII + vehicle: 8.5 ± 0.3 versus 12.5 ± 1.1 mm^2^; P=0.001). Co-infusion of AngII with amlodipine ablated AngII-induced ascending aortic dilation (8.6 ± 0.2 mm^2^; P=0.03; Figure 5). As with the suprarenal aorta, infusion of amlodipine led to preservation of a normal histological appearance of the ascending aorta (Figure 4).

### Amlodipine Reduced Atherosclerosis in AngII-infused Hypercholesterolemic Mice

**Figure 6:**
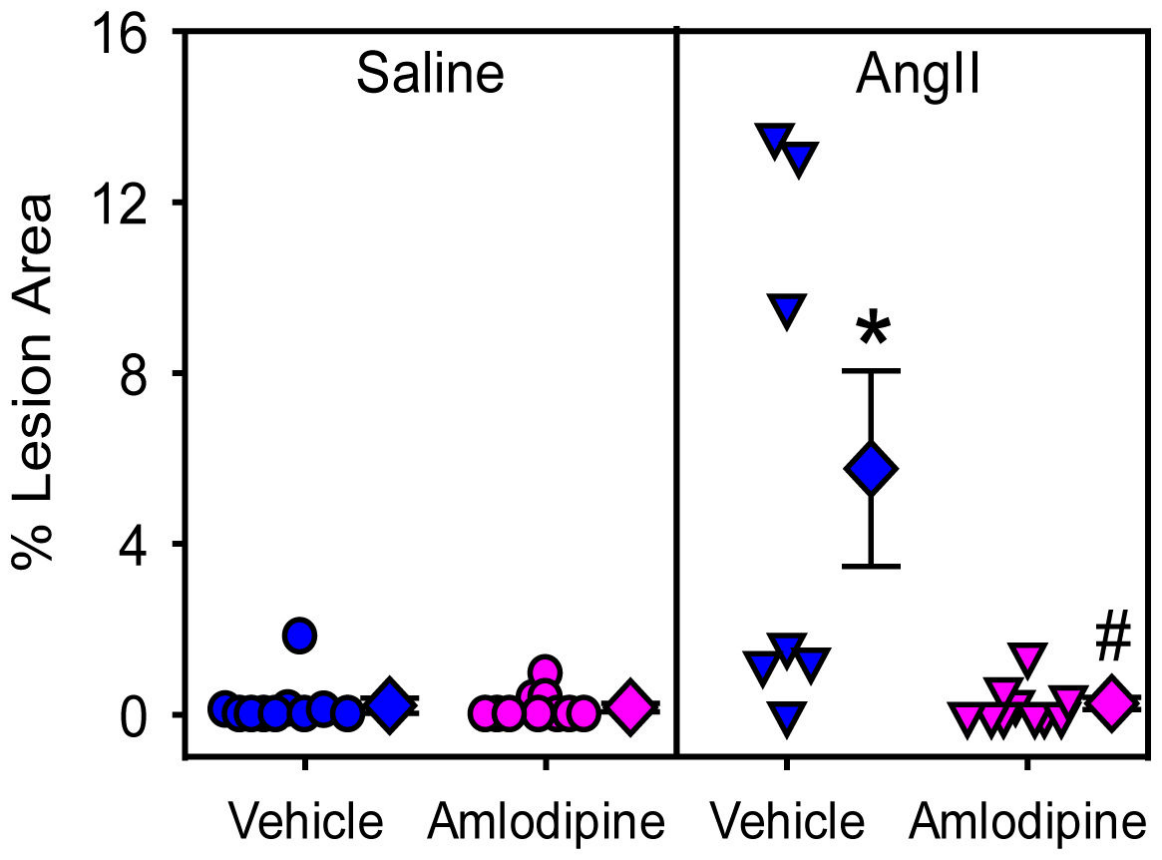
Amlodipine reduced AngII-induced atherosclerosis. Percent atherosclerotic lesion area of thoracic aortas were measured by an en face method. Circles and triangles are individual measurements, diamonds are means of the groups, and error bars are SEMs. Statistical analyses were performed using nonparametric Kruskal-Wallis test. * denotes P=0.02 for comparison between AngII + vehicle and saline + vehicle groups, and ^#^ denotes P=0.05 for comparison between AngII + amlodipine and AngII + vehicle groups.

 Atherosclerotic lesion size was only minor in mice infused with saline + vehicle and saline + amlodipine after 5 weeks of feeding the saturated fat-enriched diet. Consistent with previous studies [9,39], mice infused with AngII had significantly increased lesion area in the thoracic aorta (saline + vehicle versus AngII + vehicle: 0.22 ± 0.18 % versus 5.76 ± 2.29 %; P=0.02; Figure 6). Co-infusion of amlodipine reduced atherosclerosis in AngII-infused mice (0.27 ± 0.14 %; P=0.05). 

## Discussion

 AngII infusion simultaneously induces aneurysms in the suprarenal and ascending regions of the aorta and augments atherosclerosis in hypercholesterolemic mice, as demonstrated in the present and previous studies [9,12,14,40]. Despite markedly different vascular pathologies promoted by chronic AngII infusion, the present study provides evidence that continuous infusion of a dihydropyridine calcium channel blocker, amlodipine, has pronounced effects on inhibition of these diverse pathologies that developed in mice rendered hypercholesterolemia by deletion of LDL receptor and feeding a diet that has a high saturated fat content (42% by calories), compared to normal laboratory diet (18% fat by calories).

 Amlodipine has commonly been administrated by gavage, mixed with diet, or dissolved in drinking water [41-46]. In the present study, amlodipine was given using osmotic minipumps, as used in a previous study [47]. This approach was used to provide stable calcium channel blockade throughout the 28 days of AngII infusion. In addition to providing a mode of constant drug delivery, we also measured plasma concentrations of this drug in mouse plasma. The detected concentrations (mean concentration of saline + amlodipine and AngII + amlodipine: 29.3 ± 1.7 ng/ml) are above the reported EC50 of amlodipine [48,49]. By comparison to humans, a single dose of 10 mg amlodipine to healthy male volunteers resulted in plasma concentrations of 5.9 ± 1.2 ng/ml. Repeated daily dosing led to mean plasma concentrations of 14.5 ± 5.8 ng/ml with peaks and troughs of 18.1 ± 7.1 and 11.8 ± 5.3 ng/ml, respectively [50,51]. Therefore, plasma concentrations of amlodipine achieved in this mouse study were in a similar range to those achieved in humans. 

 Previous studies have demonstrated that increased systolic blood pressure observed during AngII infusion is not the determinant of aortic aneurysmal formation or atherosclerosis augmentation [14,40,52,53]. In agreement with these previous reports, the present study also found that amlodipine reduced all vascular pathologies without resulting in any measurable change in systolic blood pressure. This result extends the findings by Kanematsu et al. [54], who reported that amlodipine reduced both systolic blood pressure and aortic aneurysmal formation in normocholesterolemic mice. 

 We also found that amlodipine administration increased plasma renin concentrations. Previous studies suggest that amlodipine has biphasic effects on plasma renin activity in rodents, with reductions at low doses and stimulation of renin at higher doses [55]. The basis for amlodipine exerting these effects on plasma renin concentrations has not been defined. In our experience, plasma renin concentrations in mice are inversely related to plasma concentrations of AngII [24,56,57], as also demonstrated in the present study by pronounced reductions in plasma renin concentrations in AngII-infused mice. Therefore, the observed elevations in plasma renin concentration by amlodipine suggest a reduction in endogenous production of AngII. This may have contributed to the ability of amlodipine to reduce vascular pathologies.

 Although both abdominal and ascending aortic aneurysms are defined by pronounced aortic luminal expansion, pathologies in these two locations are distinct [12,22,58-60]. In AngII infused mice, abdominal aortic aneurysms appear to be initiated by rapid medial macrophage accumulation co-localized with focal elastin fragmentation, and these changes are followed by rapid luminal expansion and marked leukocyte infiltration in the adventitia [61,62]. In contrast, during the initiation of AngII-induced ascending aortic aneurysms, one of the earliest pathological changes is hemorrhage restricted to the outer medial layers, and subsequent concentric medial changes and luminal dilation, while leukocyte infiltration is not as pronounced as in abdominal aortic aneurysms [60]. In spite of significant differences of these two aortic aneurysms, the present study demonstrated that amlodipine reduced aortic expansion in both abdominal and ascending regions. In agreement with our findings, a recent study reported that amlodipine profoundly reduced incidence of abdominal and thoracic aortic aneurysms in normocholesterolemic mice co-administered either AngII or deoxycorticosterone acetate-salt with a lysyl oxidase inhibitor, beta-aminopropionitrile [54]. This model creates profound dilation of a translucent aorta from the middle of the ascending region to aortic orifice of the subclavian arterial branch. Therefore, this pathology differs from that formed during AngII infusion in which dilation is restricted to the full length of the ascending aorta and the media is thickened and opaque [12]. Despite differences in gross appearance, amlodipine markedly decreased thoracic aortic pathologies in both animal models. Currently, information from humans with ascending aortic aneurysms is limited to a small study inferring benefits in young patients with Marfan’s syndrome who were administered a non-dihydropyridine calcium channel blocker, verapamil [63].

 Calcium channel blockade has also been studied in a rat model of abdominal aortic aneurysms induced by intra-aortic infusion of elastase. Two different members of the dihydropyridine class of calcium channel blockers, azelnidipine and nifedipine, reduced AAAs formed in this model [64,65]. In contrast to these reports and what was found in the present study, a retrospective investigation reported that calcium channel blockade was associated with the presence of abdominal aortic aneurysms. However, administration of calcium channel blockers did not affect aortic dilation rate. The authors acknowledged potential compounding factors in this retrospective analysis and noted the need for prospective studies [25]. Currently, two prospective studies are evaluating effects of amlodipine on growth of AAAs in humans (NCT01425242 and NC01118520). Completion of these studies will provide insight into the validity of translating results from AngII-induced AAAs in mice to humans. 

 In contrast to the limited information on aortic aneurysms, there have been many experimental and human studies to determine effects of multiple calcium channel blockers on atherosclerosis [66]. Several of these studies have investigated effects of amlodipine in mouse atherosclerosis models with either apoE deletion or mutation. These reports provided a range of results with amlodipine reducing atherosclerotic lesion size [41,42,46,67-69] or having no effect [43-45,70]. In the present study, we used saturated fat-fed LDL receptor -/- mice infused with AngII, and found that amlodipine decreased atherosclerosis. The conflicting findings in the literature may be partially explained by different doses of amlodipine used and mode of drug delivery; however, potential mechanisms of the conflicting findings remain to be unraveled.

 The overt differences in the appearance of AngII-induced abdominal aortic aneurysms, ascending aortic aneurysms, and atherosclerosis imply that the octapeptide is stimulating several mechanisms to produce these diverse pathologies. Therefore, the profound effects of amlodipine on all these pathologies also imply that the drug is potentially interfering with many mechanisms. Amlodipine has been demonstrated to influence many processes that have been invoked in AngII-induced vascular pathologies include reductions in oxidant stress [41], matrix metalloproteinases [71], nitric oxide synthesis [72], and leukocyte migration [47]. However, the contribution of each of these mechanisms to reducing AngII-induced vascular pathologies remains to be determined. 

 In conclusion, administration of amlodipine was studied in a mouse model that simultaneous developments aortic aneurysms and atherosclerosis. Although several studies have observed disparities in response to different AngII-induced vascular pathologies, this study demonstrated that continuous infusion of amlodipine, at a dose that achieved therapeutically relevant plasma concentrations, markedly reduced all these pathologies.
